# Caffeine-catalyzed gels

**DOI:** 10.1016/j.biomaterials.2018.04.010

**Published:** 2018-07

**Authors:** Angela M. DiCiccio, Young-Ah Lucy Lee, Dean L. Glettig, Elizabeth S.E. Walton, Eva L. de la Serna, Veronica A. Montgomery, Tyler M. Grant, Robert Langer, Giovanni Traverso

**Affiliations:** a Department of Chemical Engineering and Koch Institute for Integrative Cancer Research, Massachusetts Institute of Technology, Cambridge, MA 02139, USA; b Department of Materials, University of Oxford, 16 Parks Road, Oxford OX1 3PH, UK; c Division of Gastroenterology, Brigham and Women's Hospital, Harvard Medical School, Boston, MA 02115, USA

**Keywords:** Biocompatible materials, Shape-changing thermosets, Green-chemistry

## Abstract

Covalently cross-linked gels are utilized in a broad range of biomedical applications though their synthesis often compromises easy implementation. Cross-linking reactions commonly utilize catalysts or conditions that can damage biologics and sensitive compounds, producing materials that require extensive post processing to achieve acceptable biocompatibility. As an alternative, we report a batch synthesis platform to produce covalently cross-linked materials appropriate for direct biomedical application enabled by green chemistry and commonly available food grade ingredients. Using caffeine, a mild base, to catalyze anhydrous carboxylate ring-opening of diglycidyl-ether functionalized monomers with citric acid as a tri-functional crosslinking agent we introduce a novel poly(ester-ether) gel synthesis platform. We demonstrate that biocompatible Caffeine Catalyzed Gels (CCGs) exhibit dynamic physical, chemical, and mechanical properties, which can be tailored in shape, surface texture, solvent response, cargo release, shear and tensile strength, among other potential attributes. The demonstrated versatility, low cost and facile synthesis of these CCGs renders them appropriate for a broad range of customized engineering applications including drug delivery constructs, tissue engineering scaffolds, and medical devices.

## 1 Introduction

Robust and reproducible manufacturing of biocompatible materials with advanced functional properties is necessary to support the rapid growth and evaluation of novel biomedical technologies, especially with the rise of combination devices and the need for minimally invasive surgical techniques. Successful translation from bench concepts to clinic products is often limited by the few materials available for device and formulation development that can be easily processed and quickly pass regulatory hurdles. Furthermore, few formulations and materials platforms are dynamic and diverse enough to fulfill structural, manufacturing and therapeutic needs of a product. Covalently crosslinked thermosets provide unique advantages for structural engineering by offering robust mechanical properties but often rely on complicated processing conditions using harsh solvents, high temperatures, or toxic catalysts that preclude direct implementation in biomedical applications [[1], [2], [3], [4], [5], [6], [7], [8], [9], [10], [11], [12]]. Alternatively, polymer assemblies such as hydrogels maintain dynamic interaction with *in vivo* fluids enabling bioresponsive functions such as delivery of cargo, adsorption, and scaffolding [[4], [5], [6], [7], [8], [9], [10], [11], [12], [13], [14], [15], [16], [17]]. However, intricate processing, poor mechanical properties, and low drug loading limit the structural functionality of these gel-based systems. New frontiers in biomedical engineering focus on the development of mild synthetic procedures that produce materials responsive to their application environments [[13], [14], [15], [16], [17]], especially *in vivo*, and with a particular focus on gel-based systems [[18], [19], [20], [21], [22], [23], [24]]. While these advances herald the importance of polymer innovations, none provides easily implemented methods for direct preparation and application *in vivo*, hindering the momentum and potential of this exciting field.

Motivated by the emergence of vitrimers and concepts of dynamic thermosets [[6], [7], [8], [9], [10], [11], [12]] and covalent networks [[25], [26], [27], [28]], we developed a novel class of materials within this family appropriate for accelerated and simplified translation to clinical application. Recognizing the challenges in synthesizing covalently cross-linked hydrogels and thermosets [[1], [2], [3]], we identified caffeine, a naturally derived small molecule commonly found in consumer products, as a mild base able to catalyze simple organic reactions to build dynamic networks. When added to citric acid, a multifunctional crosslinking reagent, caffeine is capable of deprotonating the labile carboxylic acid to form a nucleophilic carboxylate that can subsequently ring open reactive functionalities such as epoxides. When coupled with bifunctional monomers like diglycidyl ether functionalized oligomers, the caffeine catalyzed carboxylate ring opening of epoxies affords a modular single step synthesis platform that enables efficient high throughput evolution of materials with tunable physical, chemical, and mechanical properties (Scheme S1). The stoichiometry of the gel defines the inherent dynamic nature imparted by the presence of residual carboxylic acids from the citric acid group. For example, an equal (1:1) molar ratio of citric acid:diglycidyl ether poly(ethylene glycol) has the capacity to form a 3 dimensional fully crosslinked network with latent carboxylic acids available to catalyze bond exchange at elevated temperatures.

**Scheme 1:**
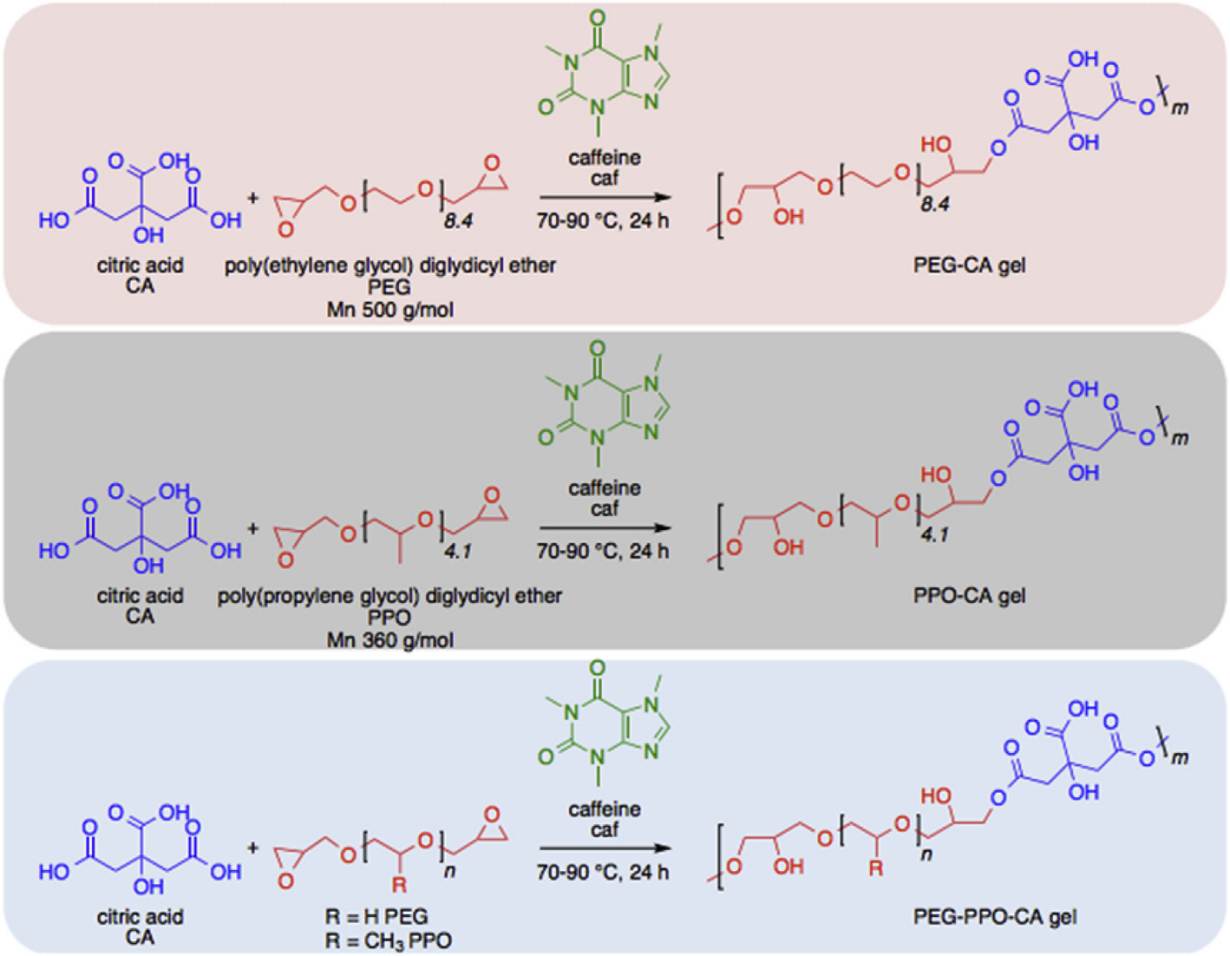
**Three variations of CCGs synthesized via caffeine catalyzed crosslinking of citric acid with different diglycidyl functionalized oligomers.** Caffeine is used to catalyze CCG formation via deprotonation of citric acid and epoxide ring opening of diglycidyl ether functionalized oligomers of PEG and PPO.

This report explores three variations of the caffeine-catalyzed gel (CCGs) concept (Scheme 1), with a focus on utilization of FDA recognized biocompatible and ingestible materials such as polyethylene glycol, polypropylene glycol and citric acid. This modular reaction platform is compatible with many combinations of multi-functional carboxylic acid/epoxy containing monomer pairs enabling diverse libraries of gels with customizable network properties. Internal gel chemistry presents opportunities for ionic or covalent association of targeted cargo with the capacity to generate higher order environmentally responsive release profiles. The synthetic conditions allow direct drug loading during manufacturing and controlled release when incubated in *in vitro* or *in vivo* aqueous environments. This process is designed to be accessible to all minimally equipped biomedical engineering labs and represents a new class of materials able to provide broad functionality in a diverse set of applications ranging from drug delivery to food science. Innovations in drug delivery and device engineering will benefit from the reproducible and robust manufacturing methods that employ readily available feedstocks to construct functional novel materials.

## 2 Materials and methods

### 2.1 Materials

Reagents and compounds from the FDA's Generally Regarded as Safe (GRAS) materials list were selected as candidates for forming bioresponsive bonds such as esters, amides, ethers, or thioethers. Additionally, well-defined, inexpensive oligomers of common biomedical engineering polymers were selected to provide an easily translatable platform applicable to a broad set of polymer feedstocks. Focusing on simple acids derived from plant and food sources such as citric, succinic, and malic acids, we used caffeine, as a mild base to deprotonate the carboxylic acids. The resulting carboxylates can ring-open epoxides to form ester bonds using solvent-free conditions.

All reagents were purchased from Sigma Aldrich unless otherwise noted and used as received. Citric acid and caffeine were stored in a desiccated environment to control moisture. Poly(ethylene glycol) diglycidyl ether (PEG, 500 g/mol), poly(propylene oxide) diglycidyl ether (PPO, 380 g/mol) were stored in sealed opaque containers in 4 °C refrigerators.

Simulated gastric fluid (SGF) and simulated intestinal fluid (SIF) were prepared as 1 L stock solutions according to US Pharmacopeia recommendations, stored at room temperature (approximately 21 °C) until use. To prepare SGF, 2 g of dry NaCl was dissolved into 1 L of deionized water. The solution was titrated using concentrated HCl to reach a pH of 1.2. To prepare SIF, 6.8 g of KH_2_PO_4_ and 0.896 g of NaOH were dissolved into 1 L of deionized water and titrated to reach a pH of 6.8. Phosphate buffered saline was prepared by dissolving PBS preparatory packet from Aldrich in 1 L of deionized water and stored as a stock solution until use.

### 2.2 Representative polymer synthesis

All reagents were directly measured into a flask equipped with a stir bar, heated to 70 °C, and well mixed to form a homogeneous viscous pre-polymer. Shortly after reaching the gel point, the viscous reaction mixture was poured into silicone molds and incubated at 90 °C to accelerate extensive cross-linking. Temperatures and times for pre-polymer formation and final gel cross-linking density were customized based on reagents and application needs. The rate of network formation can be further accelerated with higher catalyst concentration or higher heat, or by using anhydrous conditions. The ability of selected reaction conditions to form complete crosslinked networks was validated by performing an extraction on representative CCGs and evaluating residuals, ensuring only the catalyst is removed in this experiment.

For the PEG.CA gels specifically, dry caffeine (114.13 mg, 0.588 mmol) and citric acid (1.24 g, 5.88 mmol) were measured into a 20 mL vial equipped with a Teflon lined stir bar. Diglycidyl ether poly(ethylene glycol) (3.0 mL, 5.88 mmol) was added directly to the powders and the entire mixture was incubated at 70 °C and stirred at 350 rpm for 5 min until well mixed. The reaction was allowed to proceed to its gel point as observed in a rapid increase in viscosity. At this time the mixture was poured into a mold of choice and transferred to a convection oven at 90 °C. After 24 h the pieces were removed from the oven for analysis. At this point no post-processing was required as it was found all reagents were reacted into the network and no residual material was left behind. All other materials were synthesized using the same protocol as described above, maintaining molar ratios and adjusted for mass amounts.

### 2.3 Fourier transform infrared spectroscopy

Infrared spectra were recorded on an ALPHA FT-IR spectrometer (Bruker) and analyzed using OPUS v. 6.5 software (Bruker). Spectra were recorded at several time points during gel formation. Each time point represents an aliquot taken from a single continual polymerization and was directly measured on the ZnSe ATR crystal as either a liquid, gel, suspension or solid depending on the time at which the sample was measured. For example, at 15 min the polymerization was not crosslinked and still a liquid, therefore the liquid was directly dispensed on the crystal but at 600 min the mixture was a solid, thus a thin slab of polymer was directly placed on the crystal. Background spectra were recorded between samples and the crystal was cleaned with isopropanol.

### 2.4 Nuclear magnetic spectroscopy

^1^H NMR spectroscopy analysis was performed on an AVANCE 400 spectrometer (Bruker) with a 5 mm PABBO BB/19F-1H/DZ-GRD z10861810556 probe. Spectra were analyzed using MNOVA software (Mestrelab Research) and referenced in ppm to the solvent DMSO‑*d*_6_. Samples were directly removed from the polymerization and placed in an NMR tube and dissolved or swelled in DMSO‑*d*_6_ before NMR analysis.

### 2.5 Scanning electron microscopy

Surface morphology of textured materials was visualized by the means of a JEOL 5600LV (Jeol Inc.) scanning electron microscope (SEM). For visualization under SEM, samples were fixed to aluminum stubs with double-sided adhesive carbon conductive tape and subsequently sputter-coated with carbon using a Hummer 6.2 Sputter System (Anatech USA).

### 2.6 Contact angle goniometry

Fabricated surfaces were characterized for the degree of hydrophobicity by taking the static contact angle measurements by the means of the Krüss Drop Shape Analyzer DSA100 with the software Drop Shape Analyzer (Matthews, NC). Contact angles of water droplets over the sample surface, fixed to lay flat on a horizontal plane, were measured at room temperature. A fixed volume of 250 μL was dispensed onto the substrate, and then the contact angle made between the line tangent to the liquid droplet and the substrate surface was measured. The macroscopic droplet profile was photographed by a camera installed within the instrument. Each surface was analyzed in 8 different regions distributed across the plane of the surface. The average and the standard deviation values for each surface were calculated and reported.

### 2.7 Hydration kinetics

Small discs (2 mm H ⋅ 10 mm W) of PEG.CA, PEG-PPO.CA, and PPO.CA materials were incubated in simulated biological fluids (simulated gastric fluid (SGF), simulated intestinal fluid (SIF), phosphate buffered saline (PBS)) and organic solvents (ethanol, ethyl acetate, and hexanes) at room temperature (approximately 21 °C). Hydration kinetics and solvent response averages were calculated based on mass change per unit volume of representative gel formulations and represent an n = 3. For each solvent evaluated, the initial mass of a gel piece was recorded and taken as t = 0. The experiment commenced upon addition of the solvent of choice directly to the gel under study in a 15 mL conical tube. All pieces were incubated at 37 °C, rotated at 150 rpm while tilted at an angle to allow maximum mobility. At each time point the piece was removed from solution, blotted dry to remove surface water, and measured for mass immediately.

### 2.8 Mechanical characterization

Tensile, compressive, and shear properties of the polymers were characterized using standard techniques as defined by the American Society for Testing and Materials (ASTM). Testing shapes were cured by injecting viscous fluid into 3D printed molds. In all cases width, thickness, and gauge length of each sample was measured prior to testing using digital calipers. For tensile testing standard dumbbell shapes modeled after ASTM D412: 33 mm gauge length x 3 mm width x 1.5 mm thick were manufactured. Quasi-static test-to-failure (0.05 mm/s displacement rate) was conducted for n = 10 samples within each group on an Instron 5900 mechanical testing system using a 50 N load cell. The elastic modulus of samples was calculated by fitting a best-fit linear line to the stress-strain curve prior to rupture. For compressive stress-strain testing, standard cylindrical shapes molded after ASTM D695: 12.7 mm diameter x 25.4 mm height were manufactured and fitted on an Instron machine. The compressive modulus was calculated by differentiating the stress-strain curve. For shear testing standard disc shapes molded after ASTM D6204: 25 mm diameter x 2 mm thick, were manufactured. Shear stress – shear rate curves (shear strain = 5%) were generated for n = 10 samples per polymer on a TA rheometer. The shear modulus was calculated by differentiating the stress-strain curve.

### 2.9 Self-healing

To demonstrate self-healing, CCGs were ruptured at the midpoint of a dumbbell-shaped specimen as described above, through controlled tensile failure. Following rupture, the samples were placed back into PDMS molds and incubated at 90 °C in an oven for 12 h. Following self-healing the samples were stored at room temperature for 48 h prior to performing mechanical characterization. The tensile properties of specimens was determined using the quasi-static tensile test protocol described above.

### 2.10 Differential Scanning Calorimetry (DSC)

Thermal properties of CCGs were evaluated using a DSC 8000 (Perkin Elmer, USA) for 3 cooling/heating cycles from −50 °C to 180 °C at a rate of 10 °C/min with a 2 min isotherm at 180 °C. Samples were sealed in analytical aluminum pans and balanced against a reference pan with no contents. All samples evaluated were 8 mg or higher. Data was analyzed using Pyris^®^ software (version 11.0.0.0449, Perkin-Elmer).

### 2.11 LC_50_ cytotoxicity study

Polymers were completely degraded in dilute HCl pH 2. Upon confirmation of complete dissolution, the solutions were adjusted to pH 7.0 using NaOH. The final polymer solution was diluted with Dulbecco's Modified Eagle Medium (DMEM) (Life Technologies) to 50 mg/mL before testing. Cytotoxicity was tested on HeLa, HEK293, C2BBe1 (ATCC) and HT29-MTX-E12 cells (Public Health England) by seeding them in a 96-well plate at a density of 6 × 10^3^, 16 × 10^3^, 16 × 10^3^ and 2 × 10^4^ cells/well respectively. Cells were obtained directly from ATCC and were tested negative for mycoplasma. HeLa and HEK293 cells were cultured in 100 μL DMEM containing 1% non-essential amino acids, 10% fetal bovine serum (FBS) and 1% penicillin-streptomycin solution (Life Technologies) per well. C2BBe1 and HT29-MTX-E12 cells were cultured in the same medium but was additionally supplemented with 4 mg/mL human transferrin (Life Technologies). Cells were kept in culture for 3 days before replacing the medium, to which the dissolved aqueous polymer solutions were added (final concentrations of polymers ranged from 0.078 to 20 mg/mL). After 72 h, cytotoxicity was quantified by adding 10 μL alamarBlue reagent (Life Technologies) to each well. Absorbance at 570 nm was recorded on an Infinite M200Pro (Tecan) using 600 nm as reference wavelength. Following common practice, n = 8 biological replicates were recorded for each group. A positive control was provided by lysing cells with 1% Tween-20 and cells that were not subject to any polymer provided a negative control.

### 2.12 Acute oral toxicity test

In order to test *in vivo* toxicity of the polymers, we followed the guidelines for toxicity tests as outlined by the FDA [29,30]. Specifically, the FDA proposes a “limit test” for materials that are not expected to be particularly toxic. According to those guidelines, the minimum of 3 rats were dosed for each of the 3 different CCG networks discussed in a non-randomized, non-blinded experiment. Polymer samples were first pulverized to allow administration via oral gavage. Due to the large range in tensile strength, milling was not an option. Instead polymers were suspended in water, added to a commercial blender and blended for 5 min to ensure fine pulverization. The resulting slurry was frozen and lyophilized for 3 days to remove all water, yielding a fine powder. This powder was weighed and resuspended in sufficient water to obtain an injectable suspension with highest amount of polymer. For PPO.CA the maximum concentration was 340 mg/mL. PEG.CA absorbed more water and had therefore a lower maximum concentration of 170 mg/mL. Prior to administration, 3 female, retired breeder, Sprague-Dawley rats (Charles River) were fasted overnight. Suspensions were then gavaged orally at 10 mL/kg body weight. Control rats were administered 10 mL/kg water. The rats were monitored daily for 14 days with regards to symptoms of toxicity and recovery. Specifically, weight and stool consistency were measured as well as behavioral changes and any mortality were recorded daily. After 14 days, animals were euthanized by CO_2_ asphyxiation and necropsied. Thymus, heart, lungs, liver, spleen, adrenal glands, kidney, stomach, small intestine, large intestine, ovaries and uterus were extracted weighed and sectioned for histopathological examination. An independent pathologist examined all organs to detect any abnormalities in a blinded fashion. All animal procedures were conducted in accordance with protocols approved by the Massachusetts Institute of Technology (MIT) Committee on Animal Care.

### 2.13 Release experiments

Drug was added to the polymerization mixture prior to addition of the diglycidyl ether monomer. To model drug release from CCGs, analyte containing polymers were molded to 10 mm discs. n = 8 replicates were recorded for each condition. These were placed into a single fluid volume of 10 mL in simulated gastric fluid (SGF) or simulated intestinal fluid (SIF), incubated at 37 °C, and rotated at 150 rpm. Samples were immersed in 10 mL fluid each to simulate an infinite sink for elution. At each time point, 100 μL samples were removed from the stock solution and stored frozen until analysis. Release experiments were recorded as cumulative release and all concentrations were standardized against blank gel formulations to account for background interferences in the chosen detection methods. In addition, analyte stability was evaluated for each compound as a free solution of the theoretical ½ max concentration. For each 200 mg piece at a 40 wt% loading it was assumed 80 mg of compound could be potentially released. Drug concentrations were analyzed directly from the aliquoted solutions on an Infinite M200Pro (Tecan) multi well plate reader using absorbance at 345 nm for piperaquine or using excitation and emission wavelengths of 490 nm and 525 nm for FITC-dextran.

## 3 Results

**Fig. 1:**
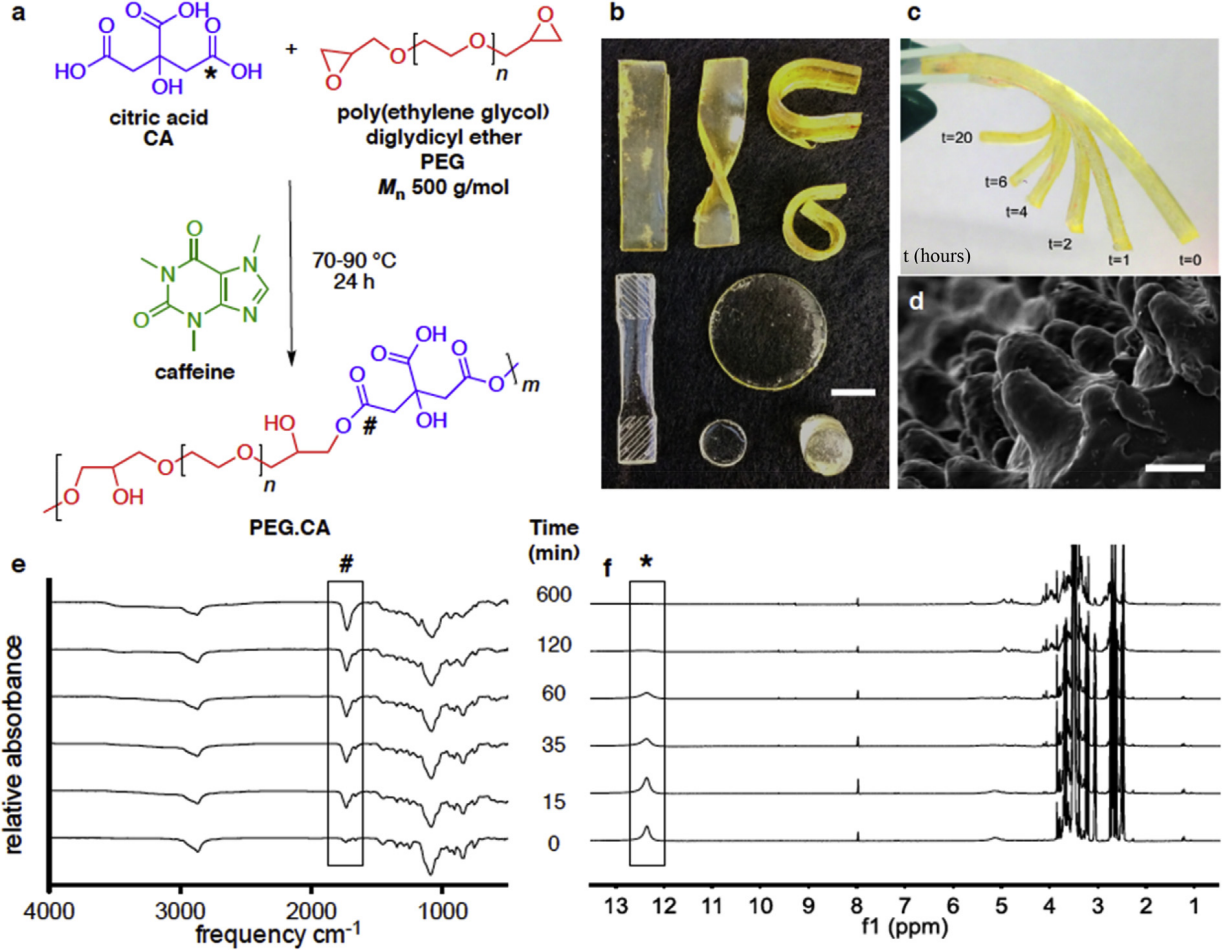
**Overview of gel synthesis and processing.** a) General one-pot synthesis of caffeine catalyzed poly(ester-ether) gels. b) Different gels shaped by casting the viscous material at its gel point into 3D silicon molds. Scale bar, 10 mm. c) Demonstration of time in hours required to fully reshape a linear rectangle into a curled object by applying external stress and incubating at 90 °C. d) SEM of a CCG surface texturized using a lotus leaf mold to mimic the micro-post structures with high resolution. Micro-posts were analyzed for 3 different classes of CCGs in 3 different sections of the molded structure. Scale bar, 10 μm. Stacked e) FT-IR of the direct polymerization mixture in liquid form and f) ^1^H NMR spectra in CDCl_3_ showing gel formation for PEG.CA via the appearance of the signature ester peak at 1755 cm^−1^ (#) and disappearance of the carboxylic acid peak at 12.5 ppm (*), respectively from t = 0–600 min of the reaction. (For interpretation of the references to colour in this figure legend, the reader is referred to the Web version of this article.)

3D cross-linked networks formed readily by using a mixture of multifunctional acids and epoxides, such as the commercially available diglycidyl ether oligomers of poly(ethylene glycol) (PEG.CA) (Fig. 1a) and poly(propylene glycol) (PPO.CA). Shapes molded out of networks containing no catalyst disintegrated in water at 37 °C reflecting insufficient covalent crosslinks to maintain robust character. Conversely, shapes molded out of networks containing 10 mol% caffeine (in relation to glycidyl-ether monomers) maintained their structure after polymerization (Fig. S1) supporting caffeine's role as a mild base capable of deprotonating carboxylic acids to initiate carboxylate ring opening of epoxides. Furthermore, these cross-linked gels did not dissolve when incubated in ethyl acetate and allowed to swell. Even though they absorbed over 20 wt% solvent, the structures returned to their original shape with only 10% mass loss after solvent removal, corresponding to loss of the associated catalyst and supporting robust matrix crosslinking. Under the same conditions, shapes containing no caffeine experienced significant mass loss (30%) when incubated in ethyl acetate and did not return to their original shape, signifying insufficient crosslinking for gel formation. Thus, all gels studied herein were synthesized with 10 mol% caffeine and cured into device shapes for 24 h to create robust constructs. Reaction conditions of 10 mol% caffeine at elevated temperatures for 24 h were selected to ensure full network formation even though lower catalyst concentration, longer reaction times and temperature are variables that can be tuned to achieve desired properties and gel formation.

The 3D devices are capable of retaining their engineered shapes at room and body temperature as determined during handling in ambient conditions by actions such as twisting, squishing and stretching (Fig. 1b). An excess of carboxylic acids from citric acid within the gel matrix ensures complete reaction of the epoxides and imparts immortal dynamic behavior with an inherent ability to catalyze transesterification reactions at elevated temperatures, a capability that can be used to reform the thermoset shapes. Reaction completion to a robust crosslinked system was validated by soaking three 200 mg pieces of each CCG in 15 mL of D_2_O for 24 h and analyzing the solution using ^1^H NMR spectroscopy to detect any soluble compounds. Fig. S3 shows that only caffeine, the catalyst, was detected, confirming complete incorporation of the monomers and supporting the corresponding mass loss calculated in Fig. S2.

CCG based materials can be reshaped by catalyzing bond exchange via applied pressure and temperature. To gauge the rate of bond exchange occurring at 90 °C, three rectangle strips of polymer from a single batch polymerization of each CCG chemistry were individually pinned in the same horseshoe configuration and incubated at 90 °C for different lengths of time (Fig. S4). Pieces were removed from the oven, allowed to cool to room temperature and retention of the adopted shape was assessed by its ability to withstand gravity and forces of manual manipulation to be unwound. A side-by-side comparison of each time point showed that 20 h is sufficient for complete reshaping to a new permanent shape (Fig. 1c). Customized surfaces with high resolution of fine features were created by using textured molds. For example, when using a negative silicone template of a natural lotus leaf, known for its super hydrophobic properties imparted by microstructures on the leaf surface, CCGs (PEG.CA, PEG-PPO.CA, and PPO.CA) showed capacity to replicate these fine micro-structures with high fidelity when evaluated under the scanning electron microscopy (SEM) and measured an increase in contact angle with water (Fig. 1d, Scheme S2).

The formation of the poly(ester-ether) networks was observed using FT-IR (Fig. 1e) and ^1^H NMR spectroscopies (Fig. 1f). Here the signature IR stretching frequency for esters at 1755 cm^−1^ increased in intensity with time signifying the formation of polyester bonds. Simultaneously a peak at 12.5 ppm in the ^1^H NMR spectra decreased in intensity over time supporting the formation of ester bonds by consumption of the carboxylic acid protons in the acid monomers.

**Fig. 2:**
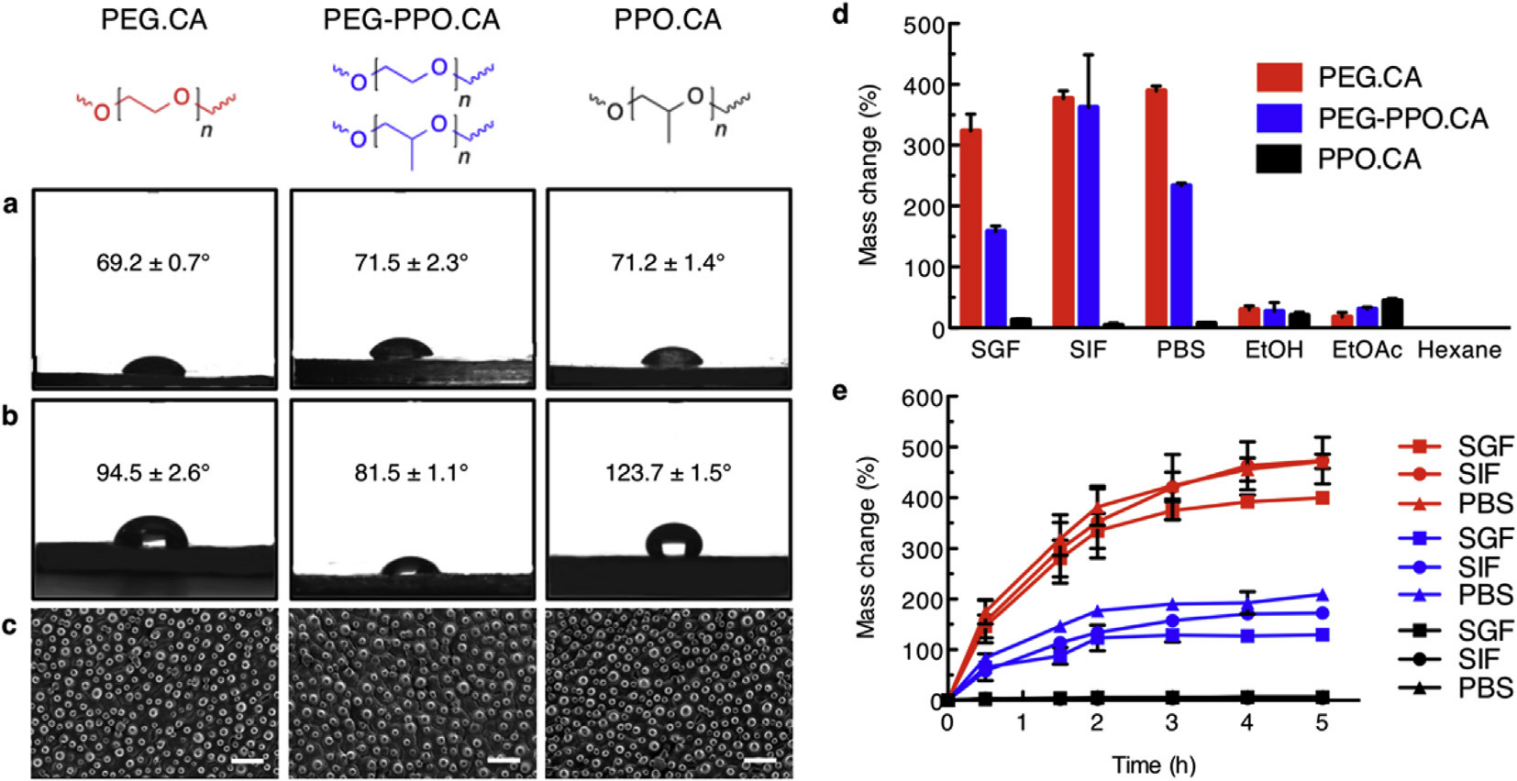
**Hydration dynamics of three chemically different CCGs.** Static contact angles (mean ± s.d., n = 8) using dH_2_O on the surface of silicon molded CCGs with **a)** no specific texturing or **b)** lotus textured surfaces. **c)** Scanning electron microscopy (SEM) images of CCGs molded on a lotus leaf textured mold. Scale bar, 50 μm. **d)** Mass change of CCGs incubated in simulated biological fluids (simulated gastric fluid (SGF), simulated intestinal fluid (SIF), phosphate buffered saline (PBS)) and organic solvents (EtOH, EtOAc, hexanes) for 24 h **e)** Hydration kinetics of CCGs in simulated biological fluids at 37 °C as measured by % mass change. In both **d)** and **e)** error bars correspond to the standard deviation of 3 samples per condition. (For interpretation of the references to colour in this figure legend, the reader is referred to the Web version of this article.)

Contact angle measurements using deionized water (dH_2_O) on the native flat surfaces of CCG samples showed little difference with regard to the oligomers used (Fig. 2a). Instead, differences in contact angles were most dramatic when gels were intentionally textured using molds that mimic the fine micro-structures of the lotus leaf (Fig. 2b). In this case the contact angle for PPO.CA materials nearly doubled, becoming significantly more hydrophobic, while the PEG.CA material increased marginally. Curiously, it was also observed that a 1:1 blend of the two polymers, PEG-PPO.CA, measured the smallest change in contact angle. Fewer micro-post structures for PEG-PPO.CA is apparent in the SEM images of the CCG surfaces and is attributed to loss of micro-posts to the mold surface upon release (Fig. 2c).

When subjecting the gels to solvents, the more hydrophilic CCGs containing PEG as oligomers not only absorbed simulated biological fluids such as phosphate buffered saline (PBS), simulated gastric fluid (SGF), and simulated intestinal fluid (SIF) extensively (Fig. 2d), but also in a more rapid fashion and showed evidence of degradation at time points beyond 5 h (Fig. 2e). On the other hand, PPO.CA based CCGs were less hydrophilic, creating CCGs that do not swell significantly nor rapidly with any solvents. The differences in SGF and SIF uptake for each of the different CCGs and subsequent network degradation is a customizable feature that can be used to enable tailored design of oral medications based on desired release kinetics. Notably, swelling kinetics could be tuned in accordance to the oligomer chemistry, correlating with differences in the hydrophobicity. This is best demonstrated in the blended PEG-PPO.CA material, where hydration kinetics showed intermediate values between the individual polymers.

**Fig. 3:**
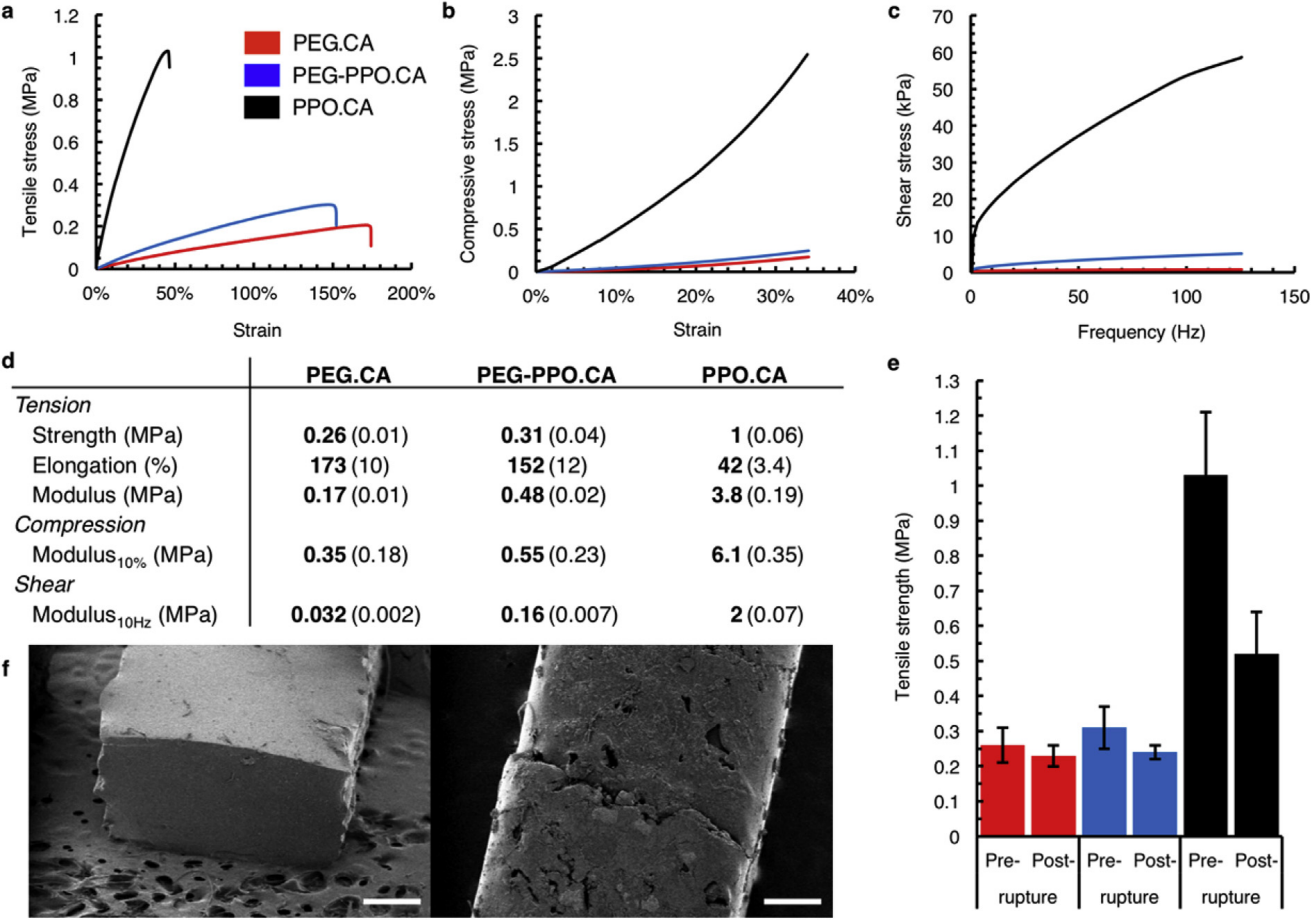
**Tensile, compressive, and shear mechanical properties. a)** Tensile test, **b)** compression test, and **c)** shear test indicating the range of mechanical properties of the CCGs and represent average values of three structures per testing condition. **d)** Summary of mechanical properties. Mean values are provided in bold and standard deviations are in brackets. **e)** Change in the tensile strength of the specimen before and after rupture test. Error bars correspond to the standard deviation of 10 samples per condition. **f)** SEM image of ruptured (left) and self-healed (right) specimen. Scale bar, 500 μm.

Tensile, compressive, and shear properties of the polymers trended in accordance to the internal chemistry of the networks (Fig. 3a–d). An increase in tensile modulus and ultimate tensile strength of the material was observed as PPO was added. Likewise, PPO has the opposite effect on the maximum elongation, which is greatest in PEG.CA. Overall, the PEG-PPO.CA co-polymer had intermediate characteristics suggesting that the chemical composition can be used to tune gel mechanical properties. Furthermore, the lack of shear thinning during tensile testing supports the complete network structure of the material behaving similar to a thermoset under these conditions.

**Fig. 4:**
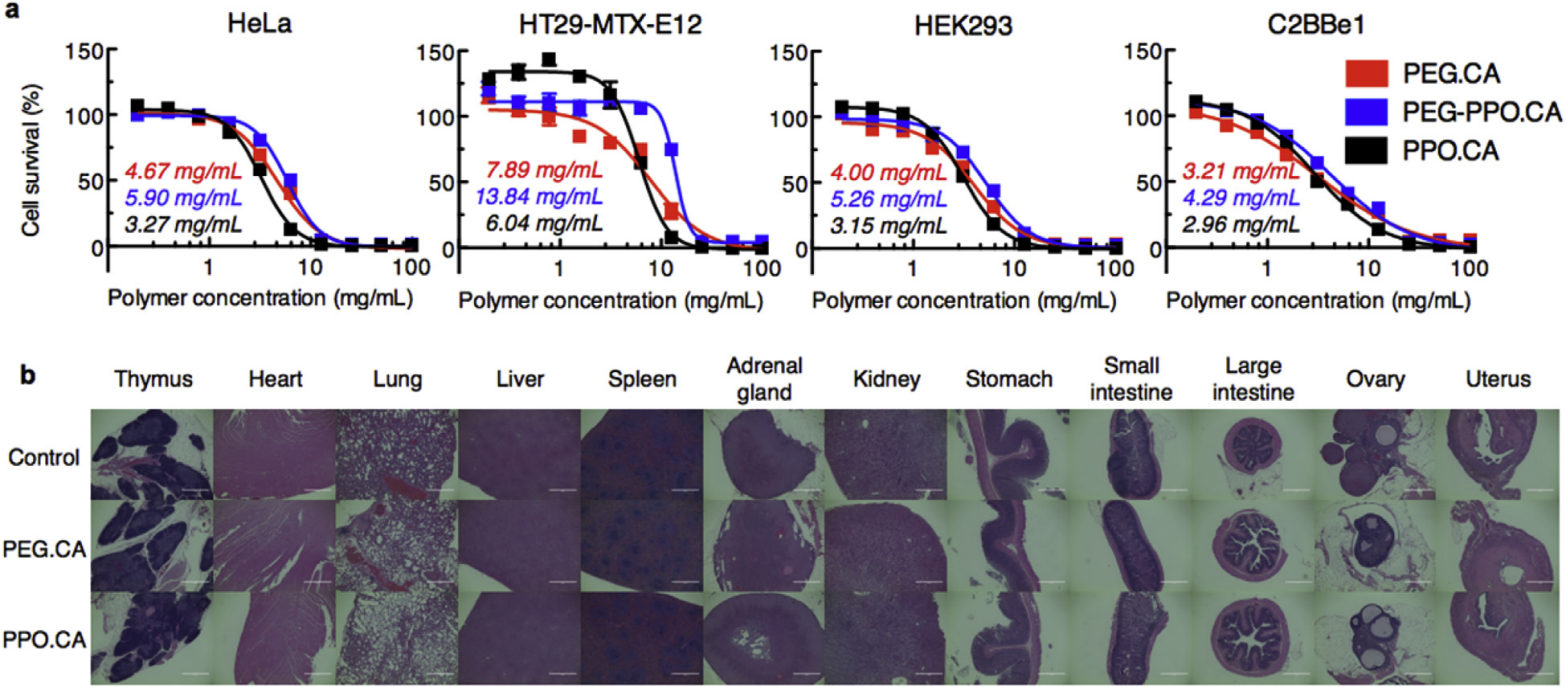
***In vitro* and *in vivo* toxicity. a)***In vitro* cytotoxicity on HeLa, HEK293, HT29-MTX-E12, and C2BBe1 cell lines. LC_50_ values are noted in the graph. Error bars correspond to the standard deviation of n = 8 biological replicates per condition. **b)** Histology of extracted organs post oral administration of CCGs to n = 3 rats per condition. Scale bar, 1 mm.

A unique feature of CCGs is the dynamic ester network with the ability to trans-esterify and impart “healing” properties to exposed interfaces with available reactive groups. This reaction is catalyzed by internal residual acidic groups on the citric acid moiety and can be catalyzed by addition of heat, consistent with findings by Montarnel et al. with a similar acid catalyzed dynamic system [6,8]. To demonstrate this concept, the same experimental setup as the tensile tests was used to stress pieces to break. After rupture, the broken surfaces were placed in contact to allow healing. First healing experiments were performed at room temperature which is above the apparent T_g_ of the CCG materials (Fig. S5), however, these conditions did not result in robust reconnection across the broken interface, even after several days. Therefore we concluded an external catalyst was required for transesterification to enable covalent bond reformation across the interface. Thus, broken CCGs were allowed to heal at 90 °C over 12 h and their tensile strength was re-evaluated using the same procedure. Rupture testing demonstrated that healing capabilities were maintained and tensile strength was restored (Fig. 3e–f). PEG.CA gels had the largest recovery in strength after being torn and healed, while the addition of PPO decreased this effect, in agreement with the lower restored strength in the parent PPO formulation. This decrease is likely due to the shorter chain length and reduced mobility of end groups in the PPO oligomers, limiting their ability to find an eligible bond for healing. Direct chemical observation of bond reformation is challenged by difficulty accessing the internal material of the healed interface, but the healing and rebreaking demonstration supports robust gel formation across the repaired interface. Furthermore, CCGs are appropriate materials for direct implication in biomedical applications as supported by *in vitro* cell toxicity showing low cytotoxicity in four cell lines (Fig. 4a). The median lethal concentration (LC_50_) was similar across all cell lines and CCG variants, ranging between 2.96 and 5.90 mg/mL. The HT29-MTX-E12 cell lines showed an even higher LC_50_ however with greater variability, ranging from 6.04 to 13.84 mg/mL, likely due to the protective mucus layer being produced by the cell lines. Furthermore, bulk *in vivo* administration of the CCGs to three rats per CCG chemistry showed no symptoms of toxicity, supporting direct use of CCGs in oral administration. In all cases test animals showed no significant weight changes, no behavioral abnormalities, and no gross or histopathological organ changes (Fig. 4b). Most significantly no deaths resulted at the maximally achievable dose of 1.7 g/kg for PEG.CA and 3.5 g/kg for PPO.CA respectively. Stool samples did not show any detectable blood, however test animals receiving CCGs did have a slightly softer stool consistency 1–2 days after administration that resembled type 4 on the Bristol stool chart.

**Fig. 5:**
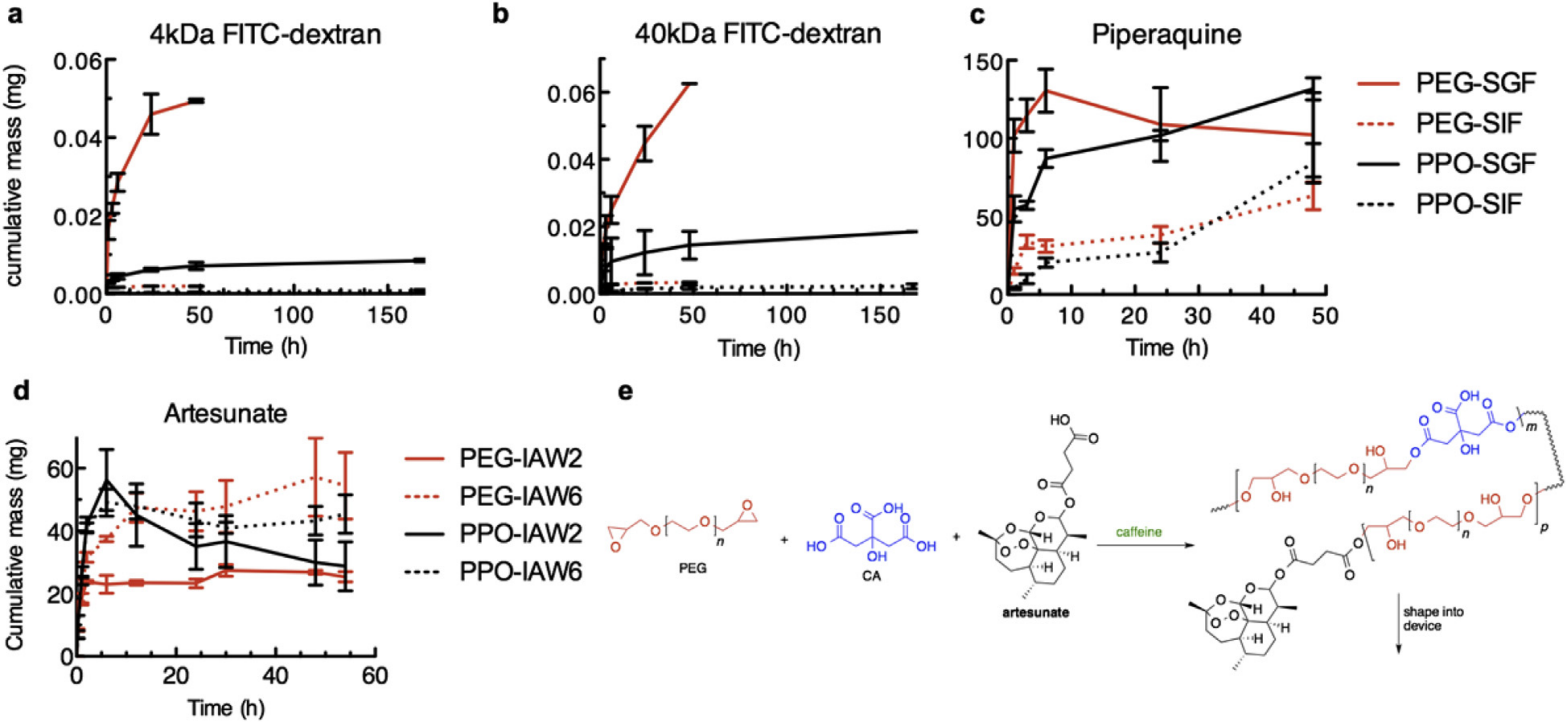
***In vitro* drug release.** Release kinetics of **a)** 4 kDa and **b)** 40 kDa FITC-dextran as well as **c)** piperaquine from CCGs into simulated gastrointestinal fluids. **e)** Release kinetics of artesunate measured in pH adjusted isopropanol/acetone/water mixture (IAW). **f)** Molecular description of proposed drug loading reaction showing potential for retention of artesunate to be covalently incorporated into the CCG network directly through a labile ester bond. Error bars correspond to the standard deviation of 3 samples per condition.

Finally, we evaluated the release kinetics of three representative compounds using UV–vis spectroscopy: 40 kDa FITC-dextran, 4 kDa FITC-dextran, and piperaquine from the two different CCGs in acidic (SGF) and alkaline (SIF) pHs each (Fig. 5). Macromolecule delivery from CCGs using FITC-labeled dextrans showed an initial burst release of material from PEG networks in SGF (Fig. 5a–b) correlating with the rapid swelling and expansion of this network, presumably permitting free diffusion and release. In agreement, the 4 kDa dextran released quicker than the 40 kDa, suggesting that internal network mobility may correlate to network crosslink density, slowing release of the larger molecule until the network is more fully expanded. The PEG.CA materials remained intact through 48 h of release testing but disintegrated thereafter, preventing further study. Alternatively, PPO.CA materials continued to release dextran beyond 150 h of data collection at a much slower rate.

The good solubility of hydrophilic piperaquine yielded a burst release for both materials (Fig. 5c). However, the less hydrophilic nature of the PPO.CA resulted in a much lower initial concentration and more gradual release thereafter. Additionally, the solubility of piperaquine in simulated biological fluids is enhanced at lower pH as reflected by the higher rate of release in SGF in comparison to SIF when piperaquine is neutral, less charged, and more likely to be retained in the polymer matrix. Finally, release of artesunate (Fig. 5d), an antimalarial compound of interest for extended release applications was analyzed in isopropanol/acetone/water (IAW) mixtures at representative pH's to simulate gastric and intestinal fluids while enabling solubility for LC-MS analysis. The reactive carboxylic acid group of artesunate presents the opportunity for direct covalent incorporation into the network Fig. 5e which requires longer release times to achieve full release of artesunate from the structure as seen in Fig. 5d where only 60% of the cargo loading is released in the same time frame as dextran and piperaquine. Overall, we demonstrate the ability to control the release kinetics of a target compound by tuning the pore size and chemical composition of our networks in comparison to the intended release fluid. Future studies will further explore opportunities to more precisely control release profiles through tailoring features of the gel including pore size, covalent attachment sites and internal chemical environments.

## 4 Discussion

Herein we introduce a simple readily accessible synthesis and engineering platform for the formation of 3D cross-linked polymer gel using caffeine as a catalyst. Time dependent FT-IR and ^1^H NMR spectroscopy experiments showed the gradual development of poly(ester-ether) networks by simultaneous formation of ester bonds and consumption of carboxylic acid protons from citric acid. This mechanism is compatible with numerous combinations of multi-functional glycidyl ether and acid containing monomers as well as inclusion of anhydrides or lactones, opening the opportunity to construct an incredibly diverse array of customized materials based on specific application needs. The simple manufacturing method with no residual solvents or toxic catalyst allow for these materials to be cast in molds of many shapes with the capacity to retain micron sized features with no measurable shrinkage or distortion and ready for immediate application, advantageous for accelerated translation and regulatory evaluation.

CCGs are stable at room temperature with the inherent capacity to be reshaped or healed representing a new class of biocompatible, dynamic thermosets that can be engineered for advanced customized applications. The shaping and healing capability is imparted by the inclusion of excess carboxylic acids in the formulation, which allow ester exchange to be easily catalyzed under heat and does not require addition of new materials or introduction of complicated interfaces, maintaining the simplicity of this platform. Shape changing or healing is time dependent and scales with the number of bonds required for rearrangement to completely relax applied stress or bridge interfaces.

Finally, hydration kinetics and mechanical testing demonstrate how the chemistry of CCGs can be tuned by changing the oligomeric backbone building blocks, empowering designer customization based on application specifications. Differences in solvent response enable the drug release profile of CCGs to be tailored with potential to further customize by including chemically diverse crosslinking monomers that feature groups capable of advanced environmental response such as ionic coordination, or disulfide bond formation. Impressively, the mild chemistry used for synthesis of CCGs permits direct loading of compatible active ingredients at the onset of device formation, providing a reproducible, efficient manufacturing method for high cargo loading. Given the highly tunable release properties, evidence of low cytotoxicity on multiple cell lines *in vitro* as well as no detectable *in vivo* toxicity after large dose oral administration in rodents we believe this platform will be useful in high-throughput evaluation of cast moldable biomedical devices intended for therapeutic applications.

## 5 Conclusion

In summary, we've demonstrated a method using caffeine and simple polyester chemistry to synthesize biocompatible covalently cross-linked gels in a one-pot system. These CCGs represent a versatile synthesis platform utilizing simple modular chemistry that can be tailored in accordance to application needs to provide broad functionality in a diverse set of applications ranging from drug delivery to food science.

## Appendix A Supplementary data

mmc1 mmc1
