# Supplementary material for: Caffeine-catalyzed gels
Source: Biomaterials. 2018 Jul;170:127–35. doi: 10.1016/j.biomaterials.2018.04.010 (PMC5937912; doi:10.1016/j.biomaterials.2018.04.010)
Supplement: mmc1 [file mmc1.docx]

**SUPPORTING INFORMATION**

**Caffeine-catalyzed gels**

*Angela M. DiCiccio^1^, Young-Ah Lucy Lee^1^, Dean L. Glettig^1^, Elizabeth S. E. Walton^1,2^, Eva de La Serna^1^, Veronica A. Montgomery^1^,Tyler M. Grant^1^, Robert S. Langer^1^, Giovanni Traverso^1,3^*

*
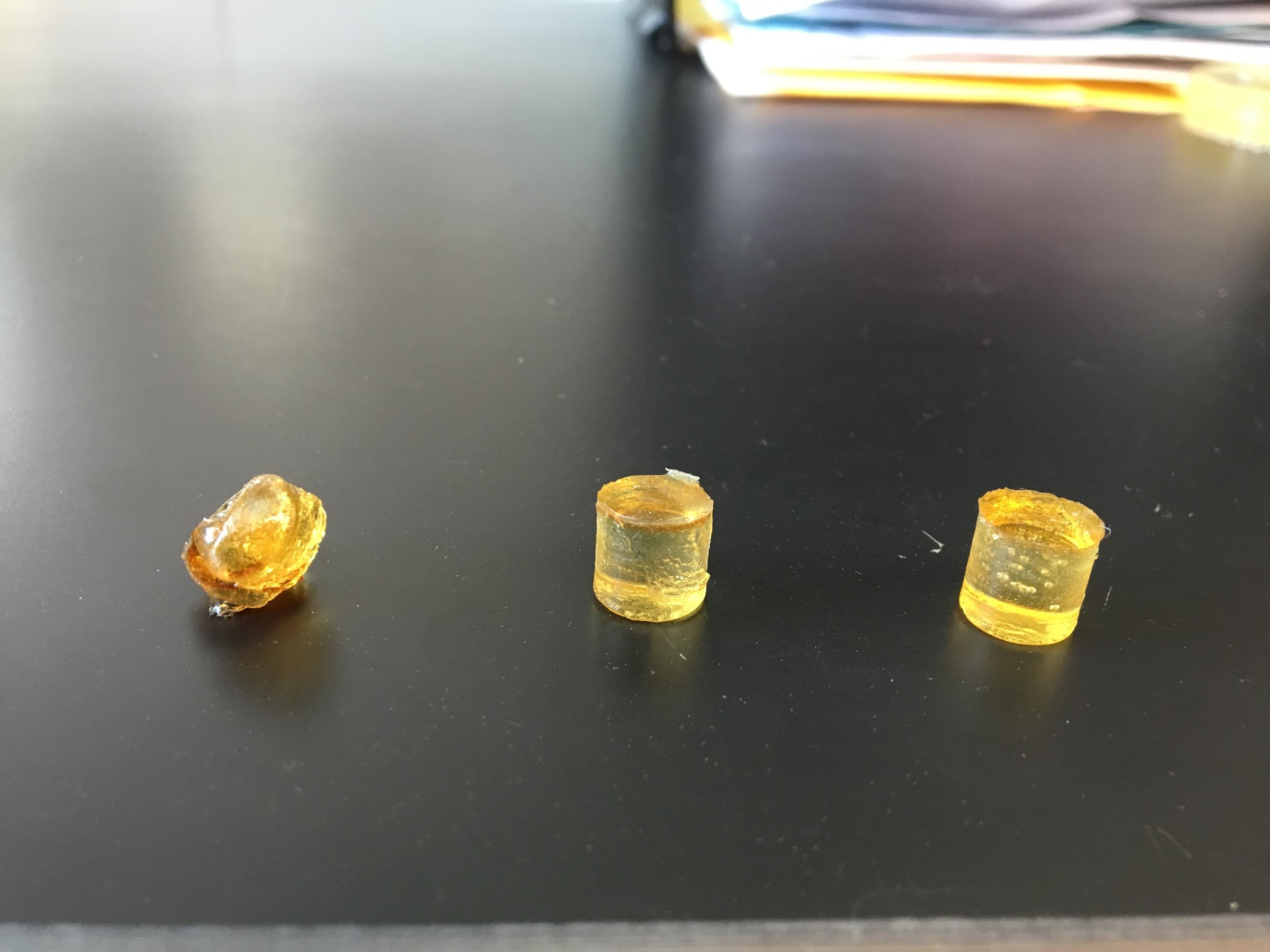

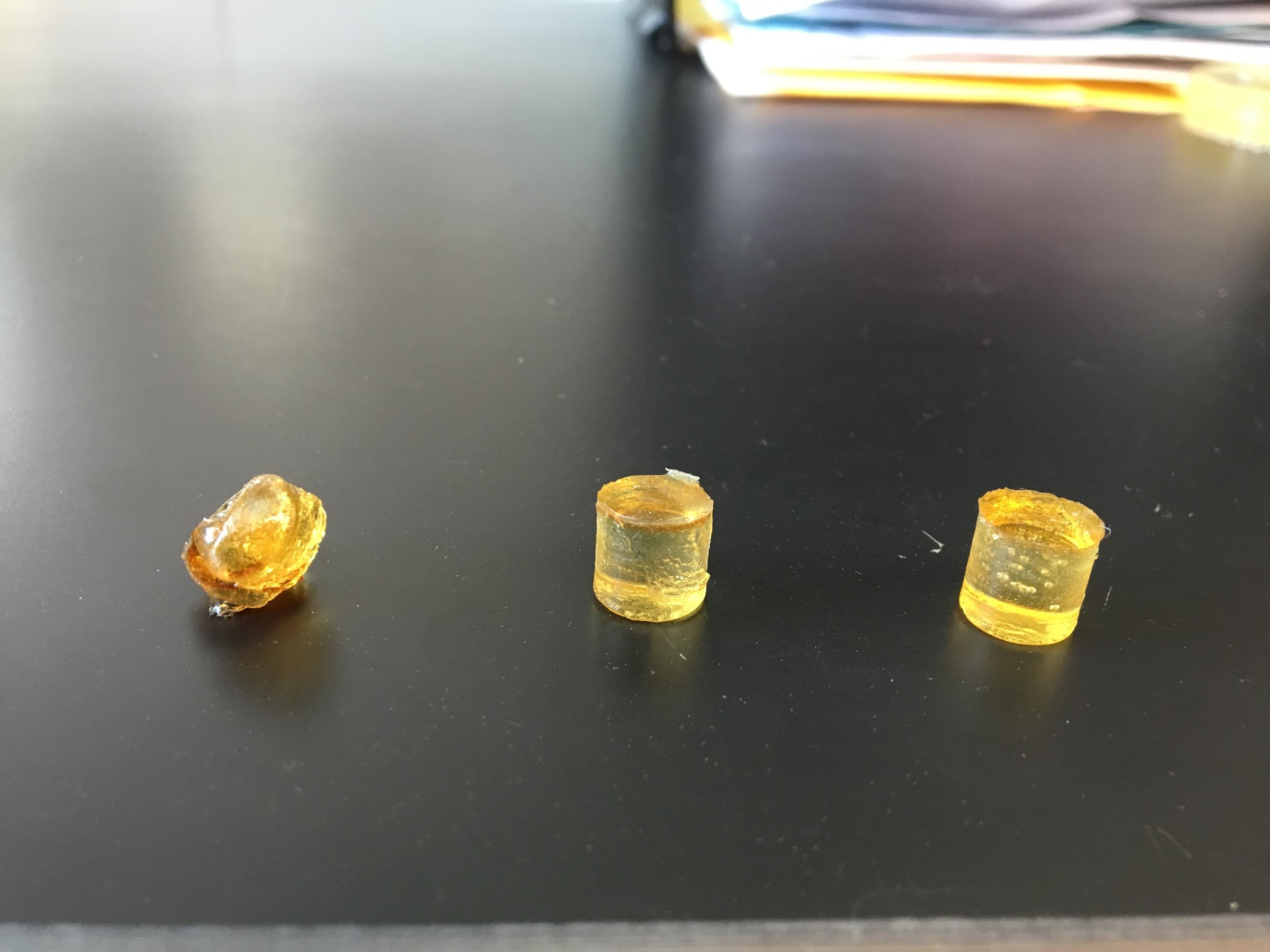
*

**Figure S1.** **Comparison of polymer structural integrity based on % catalyst used in synthesis. A comparison of network integrity for materials formed using the same process as discussed in the methods, minus a difference in catalyst concentration.** Shapes molded out of networks containing 0 mol% caffeine catalyst (left) were not able to retain structural integrity when handled at ambient temperatures. This is consistent with the low T*_g_* of PEG dimethacrylate and lack of structural reinforcement provided by network crosslinking. In comparison, networks containing 10 mol% caffeine catalyst showed strong structural retention indicating a thermoset crosslinked structure capable of retaining three dimensional structure at temperatures about the inherent T*_g_* of the constituents. Required catalyst loading for structurally stable gels was evaluated in three separate experiments for sample sizes of >3 cylinder gels in each experiment.


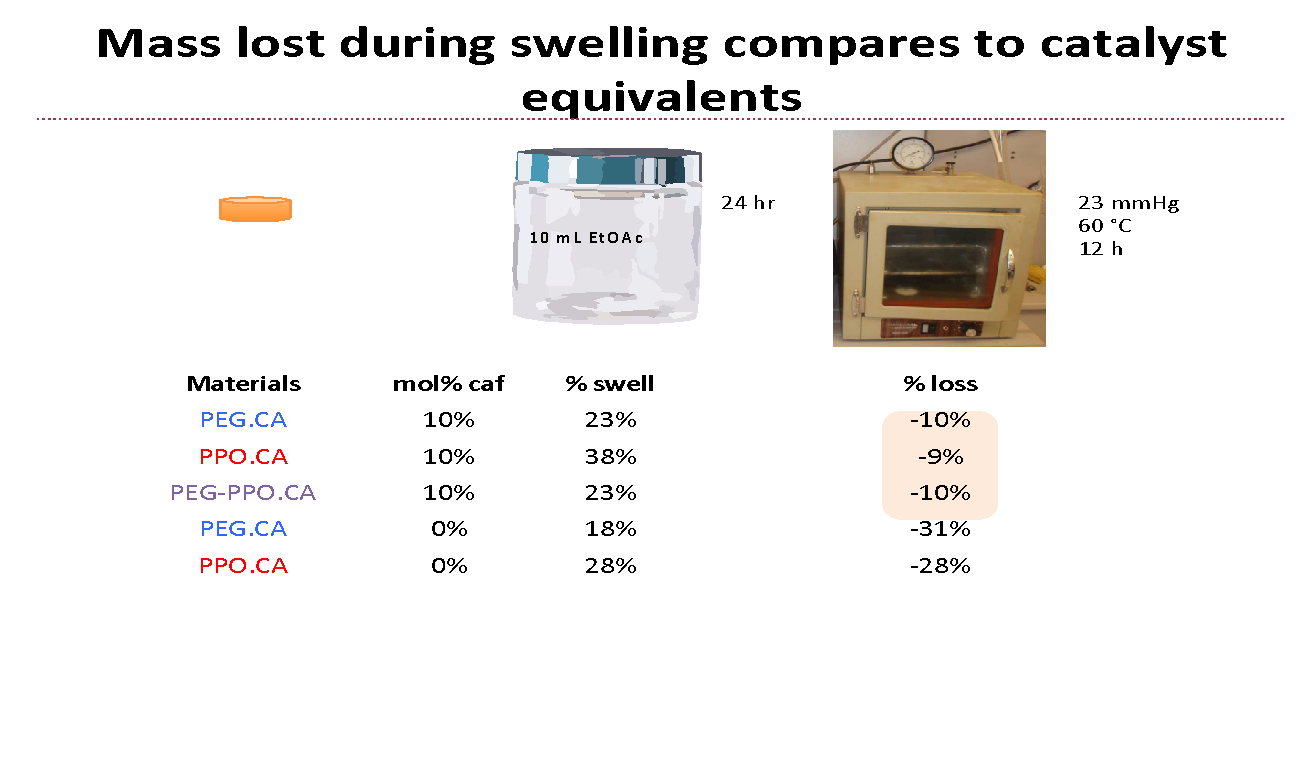


**Figure S2.** **Mass lost during extraction of cured CCGs comparing networks made with and without catalyst.** Mass loss analyzed from CCG networks after extracting with a ‘good’ solvent that is capable of dissolving each individual component of the matrix that is not part of the full network. CCGs synthesized with 10% caffeine (caf) catalyst show 23-38% mass uptake of solvent and lose 10% mass upon drying. The mass lost is equivalent to that of the catalyst, supported by NMR analysis of the residual material and the network returns to its original size and shape evidencing no structural change to the network framework itself. Conversely, networks synthesized with 0% catalyst lose about 30% mass after drying and do not retain their original structure, reflecting significant structural loss from the matrix components and incomplete crosslinking.

**
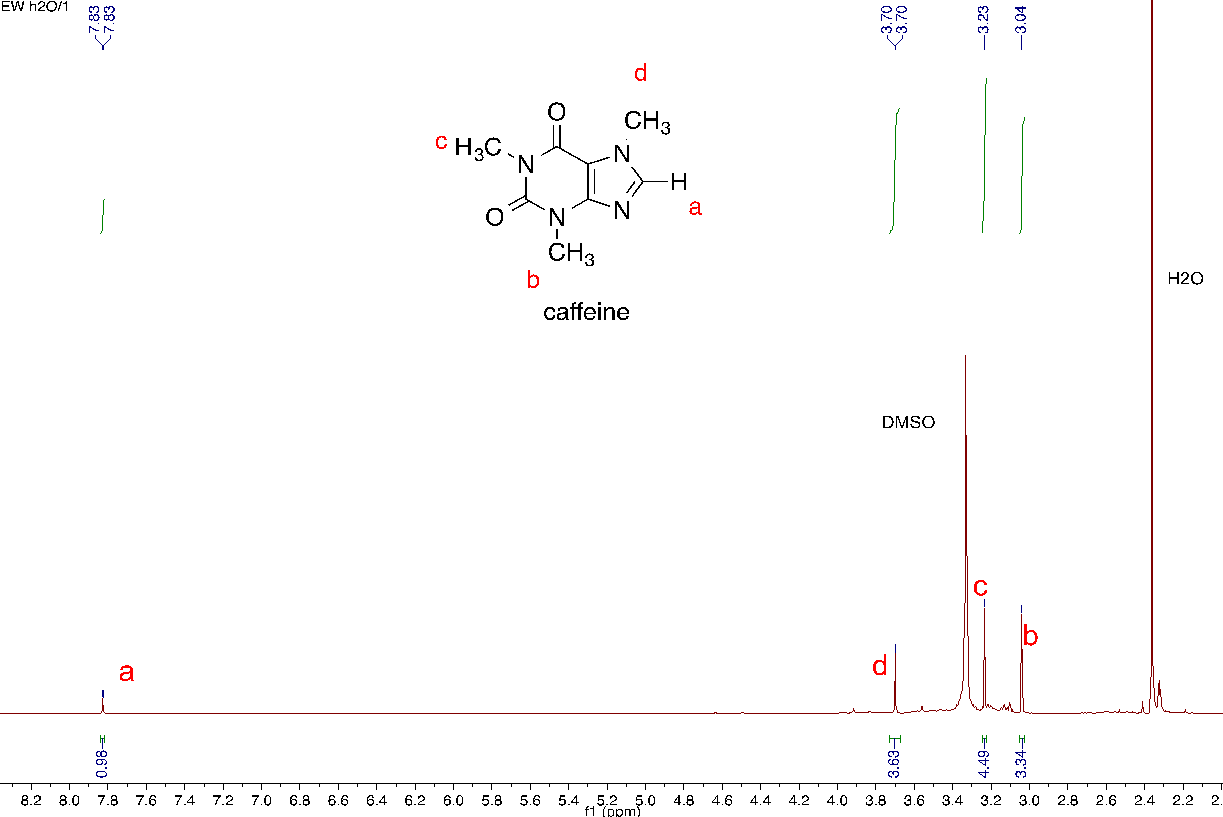
**

**Figure S3. ^1^H NMR spectra generated by the residue collected post extraction studies.**^1^H NMR spectroscopy was used to assess what components might be leached from CCGs prepared as described. Only caffeine was detected as confirmed by the peaks at ~7.8 ppm and 3-3.7 ppm. This spectrum is representative of three separate extractions of individual gels for each of the chemistries discussed in the manuscript.


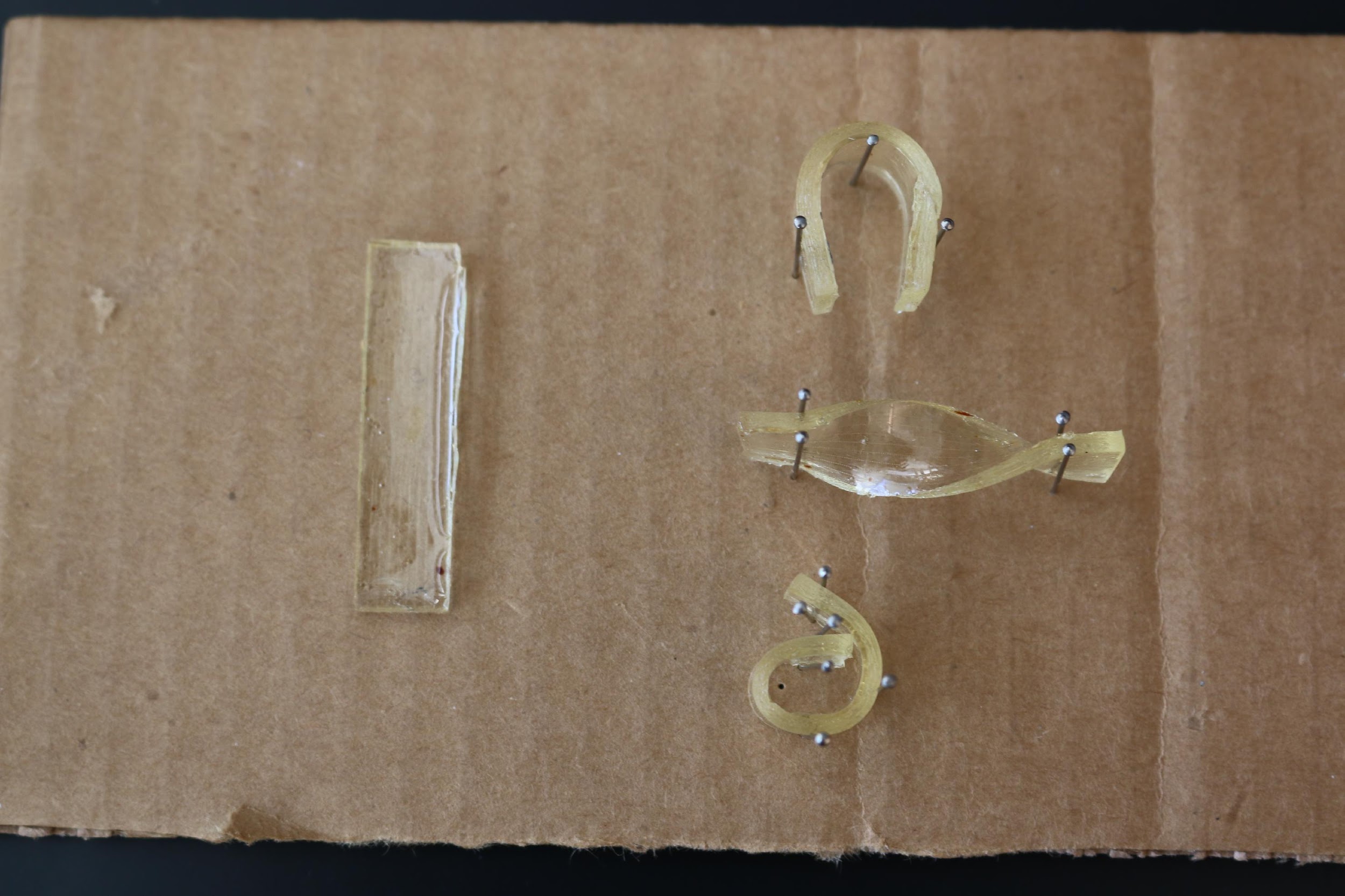
 **
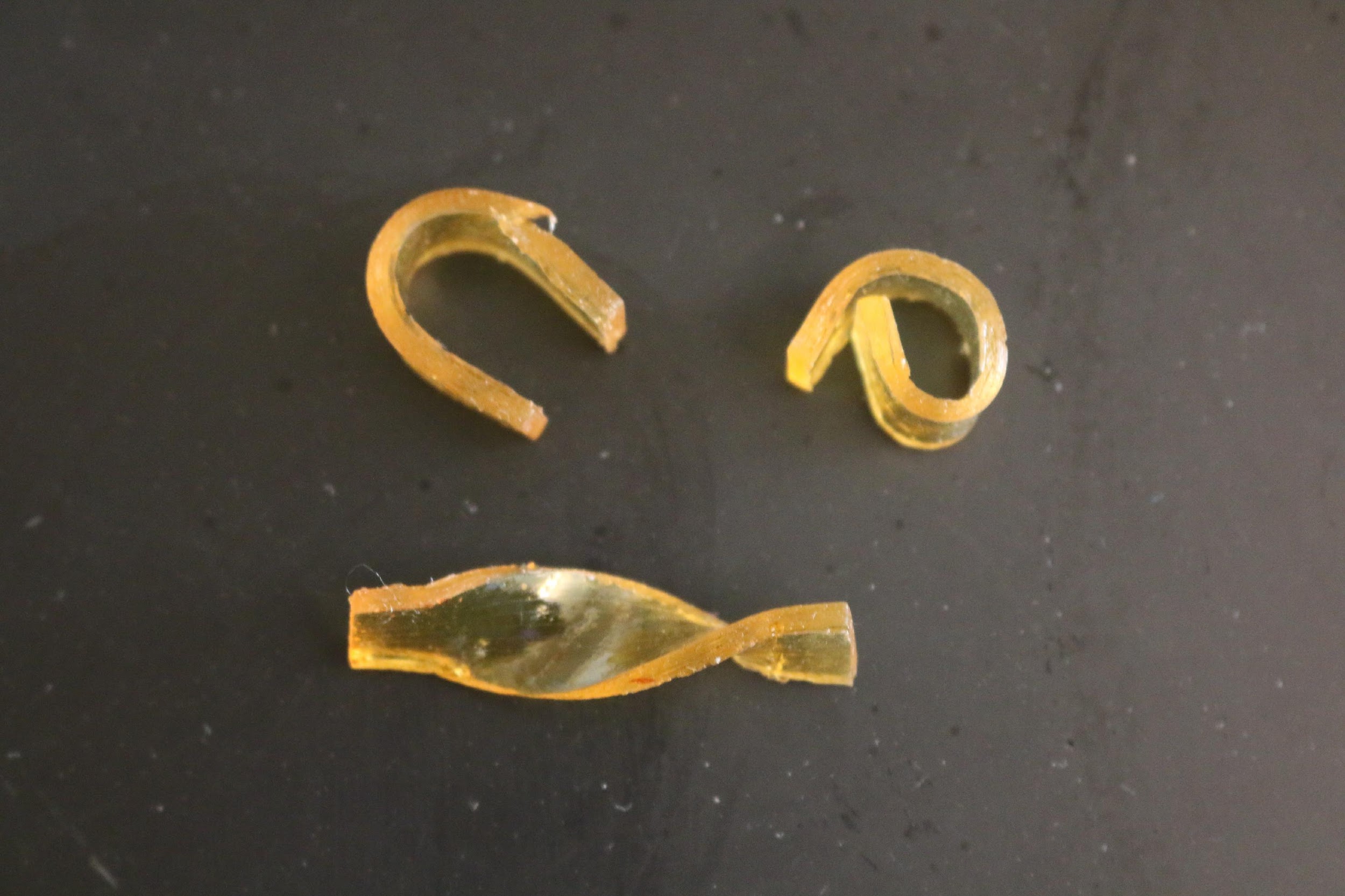
**

**Figure S4.** **Reshaping of CCGs via temperature-catalyzed transesterification under physical stress resulting in new shape formation.** Rectangle strips of polymer were pinned into different configurations (left) and heated to 90 °C. Samples were removed from the heat and their holders at different time points until final shape was securely retained (right), Reshaping capabilities were evaluated for >10 samples of each kind of CCG discussed in this manuscript with three different ovens. Each oven was used for at least 3 samples of CCG material.


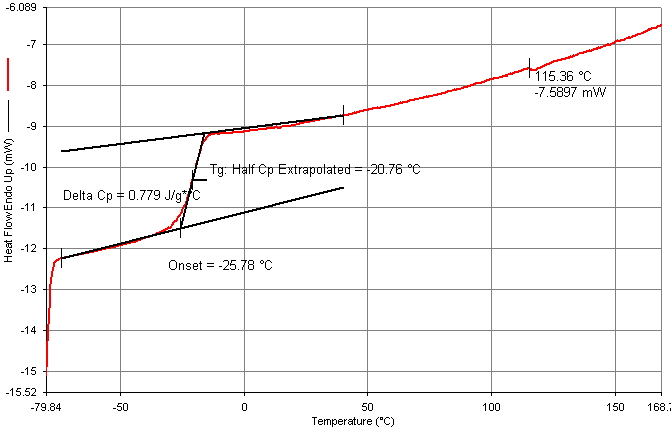


**Figure S5. DSC analysis of representative PEG CCG material showing T_g_ below room temperature.** Samples of PEG based CCG were measured into DSC pans and evaluated for phase transitions between -80 to 170 °C at a heating rate of 20 °C/min. A distinct glass transition temperature well below room temperature (-20.76 °C) is apparent, consistent with the short chain oligomer precursors used to synthesize the gels, supporting that the structural integrity of CCG’s at temperatures above their T_g_ is due to extensive crosslinking of these oligomer segments into a gel network. Note the blip at 115.36 °C is an artifact incurred during measurement.

**
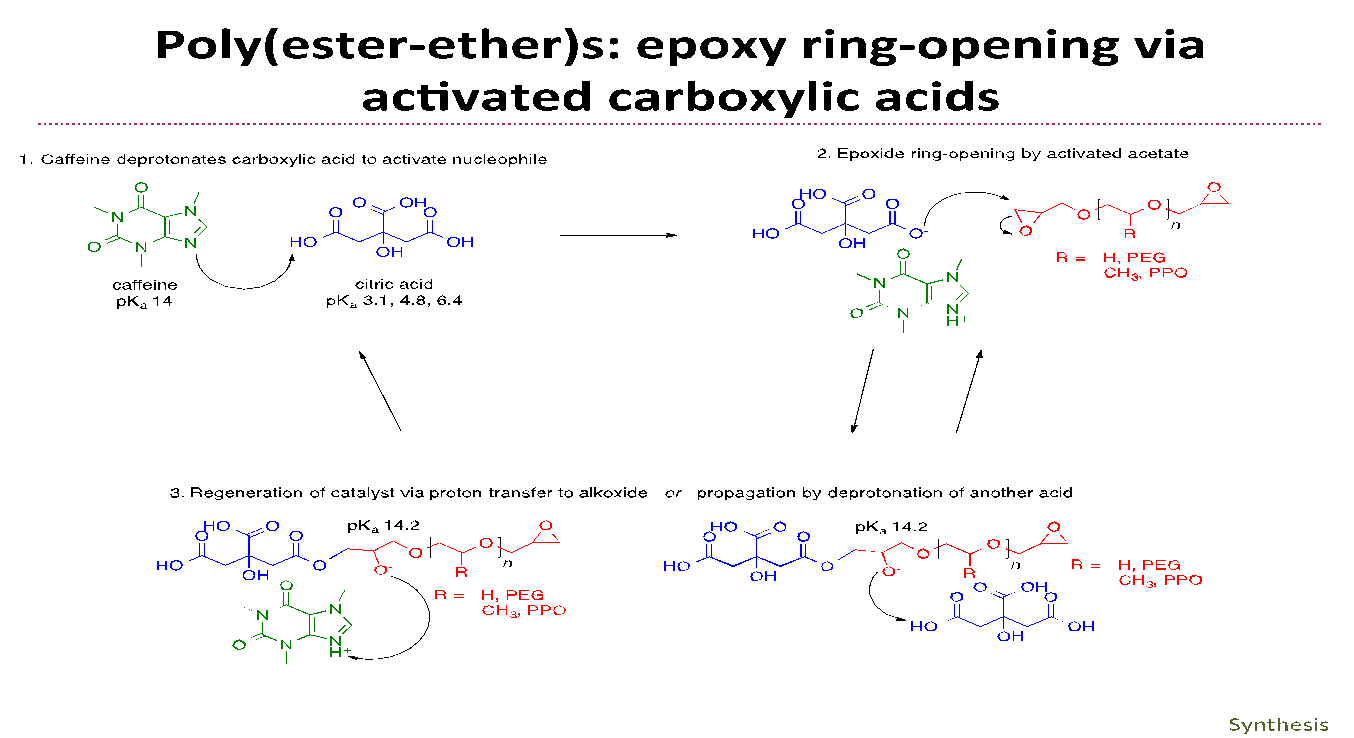
**

**Scheme S1. Proposed mechanism for formation of CCG networks via caffeine catalyzed esterification.** The mechanism of CCG polymerization begins with 1. Carboxylate activation via deprotonation of citric acid by caffeine to generation a nucleophile capable of epoxide ring-opening (2) to form an ester bond and further propagation (3).

**
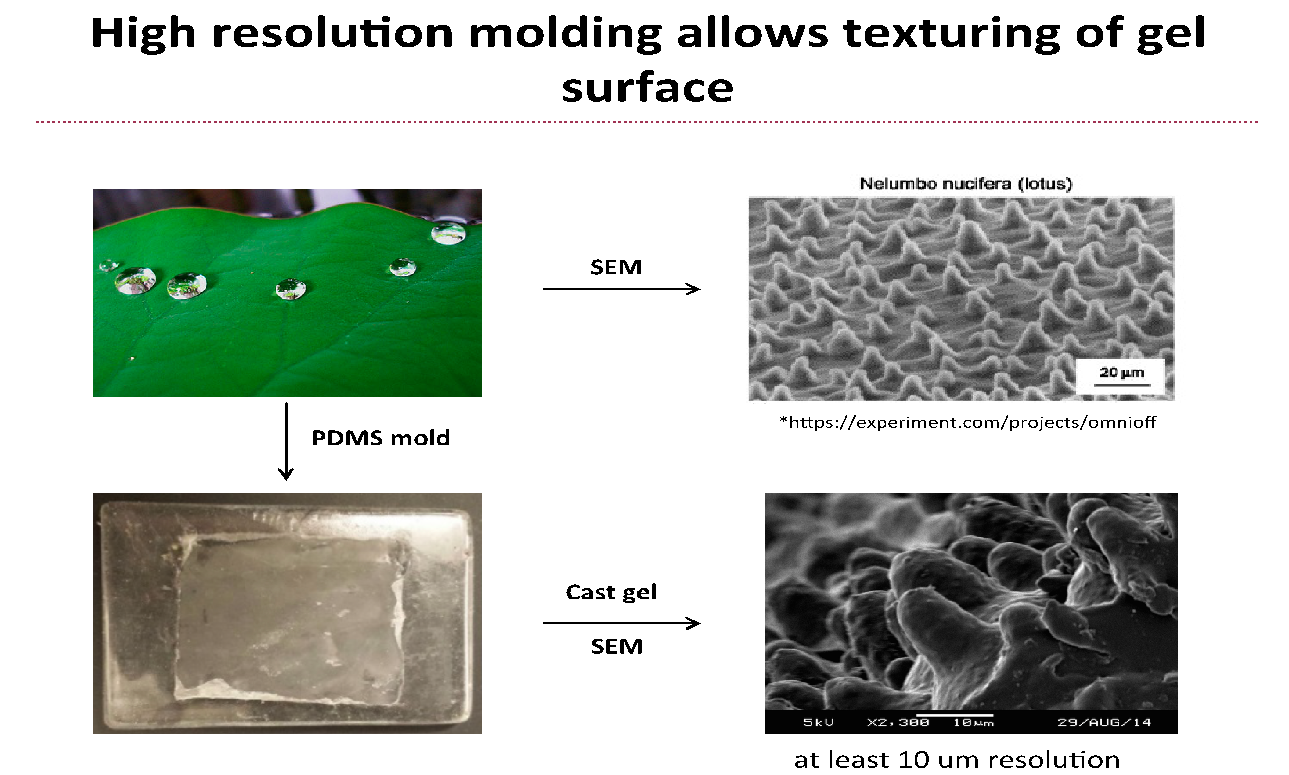
**

**Scheme S2. Visualized process for creation of lotus leaf negative molds, casting molding and replication of natural micro-structures by CCGs.** Formation of a lotus leaf negative mold via PDMS imprinting provides a mechanism for formation of lotus leaf patterned CCGs via cast molding. Replication of the lotus leaf natural micro-structures by the CCGs are visualized via SEM.


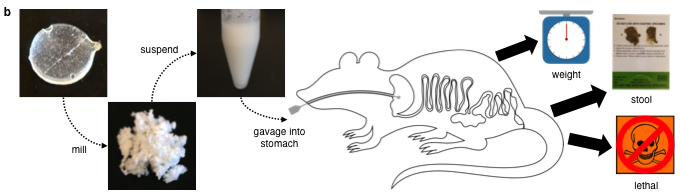


**Scheme S3.** **Visualized steps for oral dosing experiment in rats.** Outline of *in vivo* toxicity assay, showing preparation and observational outcomes of the limit assay. 3 rats each were dosed for each of the 3 different CCG network chemistries studied in the publication.
